# Unique Palliative Care Challenges in a Young Adult With a Rare Malignancy

**DOI:** 10.7759/cureus.88897

**Published:** 2025-07-28

**Authors:** Eric M Bonar, Linda S Nield, Katherine A Hill, Patrick J Tomboc, Katherine B Seachrist

**Affiliations:** 1 Department of Family Medicine, West Virginia University School of Medicine, Morgantown, USA; 2 Department of Medical Education, West Virginia University School of Medicine, Morgantown, USA; 3 Department of Surgery, West Virginia University School of Medicine, Morgantown, USA; 4 Department of Medicine, West Virginia University School of Medicine, Morgantown, USA; 5 Department of Pediatrics, West Virginia University School of Medicine, Morgantown, USA

**Keywords:** home infusion, hospice and palliative care, palliative care service, palliative endoscopic procedure, palliative interventions, tumor-associated hypoglycemia, young adult oncology

## Abstract

The incorporation of a palliative evaluation and care plan development for patients with serious illness is becoming a standard of care in modern medicine. For patients with complex challenges, palliative care physicians are well trained to offer specialized treatment plans and interventions to mitigate symptoms. This case presents the challenges of a mixed hepatocellular carcinoma and hepatoblastoma initially diagnosed in a 17-year-old male. Despite attempts at aggressive cancer-directed treatment, disease progression prompted referral for a palliative evaluation at age 20. Challenges unique to this case include malignant gastric outlet obstruction and refractory hypoglycemia. Through fostering a strong relationship with the patient, our team was able to identify his values and facilitate an individualized palliative and hospice care plan, which included endoscopic ultrasound-guided gastrojejunostomy and continuous at-home dextrose infusions. This case highlights the advancements of palliative medicine in the context of procedural and home intervention, enabling the care team to align the patient’s values with his end-of-life goals.

## Introduction

The benefit of integrating routine palliative care into oncology care has been shown to improve outcomes for patients and caregivers. This case describes how early palliative care involvement helped the team navigate uncommon challenges of advanced malignancy, utilizing unique values-based interventions. Frequent assessment of the physiologic and anatomic relationships of the patient's pathology to their symptoms is critical to develop, implement and adjust an effective treatment plan.

Primary malignant liver tumors are uncommon in the pediatric population, accounting for 1 to 2% of all cancers [1-3]. Hepatoblastoma (HB) is the most common liver tumor in children, and unlike in adults, hepatocellular carcinoma (HCC) is extremely rare in children [4]. This case presents a patient with a subtype of pediatric hepatocellular carcinoma mixed tumor with features of HCC and HB, referred to as hepatocellular neoplasm, not otherwise specified, or HCN-NOS. The epidemiology of this subtype is not readily available with current databases [5]. Literature regarding the role of palliative care specific to patients with HCN-NOS or other hepatic malignancies is minimal [6,7].

The symptoms and complications of a hepatic malignancy are similar among age groups, as well as those with other cancers. Fatigue, pain, decreased appetite, psychological changes and adverse effects of treatments are among the most common reasons for palliative care. For our patient, symptomatic hypoglycemia was one of the most challenging late sequelae of his malignancy. Non-islet cell hypoglycemia (NICH) is a recognized neoplastic complication associated with hepatic tumors, most commonly HCC, although overall rates are not widely published [8,9].

Our case highlights the ability of a diverse interdisciplinary team to deliver a unique care plan for a challenging symptom profile, to achieve the patient and family's goals. In the field of hospice and palliative medicine, invasive interventions are not routinely performed, and the use of continuous home infusions can pose significant challenges to the hospice team and families. This case demonstrates the utility of an endoscopic procedure to relieve symptoms of a malignant gastric outlet obstruction, as well as utilizing hospice services to provide at-home dextrose infusions. These interventions maximized the patient's quality of life and time spent at home with family.

## Case presentation

In light of limited treatment options, previously failed therapeutic interventions and rapid functional deterioration, a 20-year-old male, diagnosed with HCN-NOS at the age of 17 years, was referred to our outpatient palliative care clinic. Despite the initial success of an extensive surgical resection and chemotherapeutic treatment, the patient faced multiple recurrences of the disease requiring varied interventions such as hepatic transarterial radioembolization (TARE-Y90) and transarterial chemoembolization (TACE). At the time of referral, he was found to have extensive pulmonary metastasis and was started on palliative chemotherapy. 

We first met the patient and his family in the outpatient setting to establish a trusting relationship, at a time in which he was clinically stable without any imminent decisions pending. During subsequent outpatient visits the patient made it clear that maximizing his out-of-hospital time was of utmost importance as he enjoyed hunting, fishing and spending time with his animals at home. He also wished to continue interventions outside of cancer-directed treatment that would allow him to live longer. Seven months after our team’s first contact, the patient was hospitalized with a pain crisis, delirium, and tumor progression. Our team facilitated the multidisciplinary discussion with the patient and family about his cancer progression and the lack of further available cancer treatments. At a time of despondency, the patient's clear and consistent values that had been discussed many times prior allowed for a smooth transition to a comfort-focused, home-based plan. A home hospice plan was created and implemented within 36 hours after his arrival to the emergency department. 

**Figure 1:**
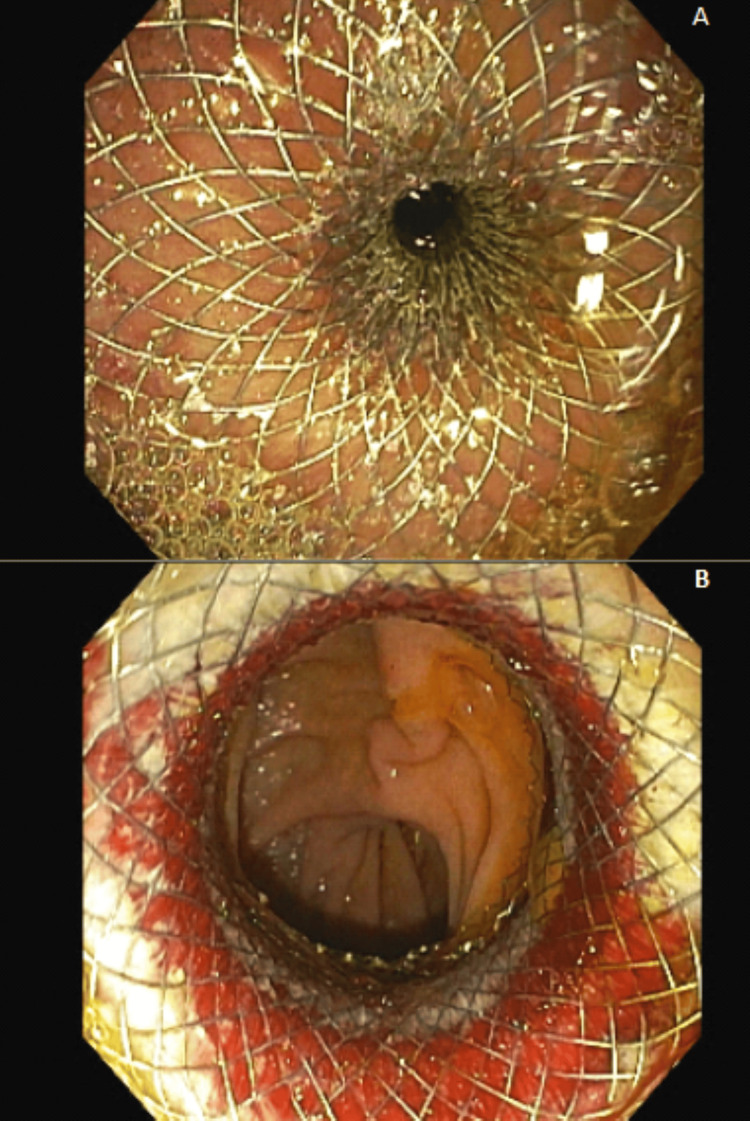
Palliative Endoscopic Ultrasound Guided Gastrojejunostomy (EUS-GJ). Lumen-apposing metal stent placed during EUS-GJ in the patient (A) providing relief from the malignant gastric outlet obstruction post-dilation (B).

While in hospice care, the patient had severe nausea and vomiting, seizure-like activity and altered mental status, prompting the family to seek care in the emergency department. Upon presentation, the patient’s blood glucose levels ranged from 30 to 50 mg/dL with multiple other lab abnormalities (Table 1). He was febrile, tachycardic, and disoriented, and was treated with antibiotics and continuous IV infusion of 10% dextrose/multiple electrolyte solution. Computed tomography (CT) imaging revealed a malignant gastric outlet obstruction (MGOO) due to extrinsic compression of the pylorus from both the hepatic tumor burden and porta hepatis lymphadenopathy. In talking with our patient, he was willing to undergo surgery or procedures to allow him to "eat real food". The palliative care and oncology teams collaborated with gastroenterology, as well as a general surgeon who is also board certified in palliative medicine, to determine the next treatment steps. To alleviate his MGOO, a palliative endoscopic ultrasound-guided gastrojejunostomy (EUS-GJ) versus duodenal stenting was ultimately recommended and performed, as he was a very poor surgical candidate, but stable for a minimally invasive endoscopic procedure. Endoscopy revealed complete gastric outlet obstruction with untraversable stenosis due to extrinsic compression on the gastric antrum and pylorus, thus EUS-GJ was completed in favor of duodenal stenting. This endoscopic procedure created a bypass between the stomach and jejunum with a lumen-apposing metal stent (LAMS) to alleviate the obstruction (Figure 1A, 1B). Our patient was able to tolerate a regular diet and was evaluated by the palliative care team, eating his mom's roast beef sandwich four days after the procedure and discharged home on postoperative day five.

**Table 1:** Labs Upon Presentation to the Emergency Department POC: point-of-care, WBC: white blood cell, RBC: red blood cell, Hgb: hemoglobin, Hct: hematocrit, MCV: mean corpuscular volume, BUN: blood urea nitrogen, ALT: alanine aminotransferase, AST: aspartate aminotransferase, INR: international normalised ratio

Lab	Result	Reference Range (units)
POC Glucose	49 (LL)	70 - 105 mg/dl
WBC	17.5 (H)	3.7 - 11.0 x10ˆ3/ul
RBC	3.61 (L)	4.50 - 6.10 x10ˆ6/ul
Hgb	10.3 (L)	13.4 - 17.5 g/dl
Hct	29.9 (L)	38.9 - 52.0 %
MCV	82.8	78.0 - 100.0 fl
Platelets	248	150 - 400 x10ˆ3/ul
Sodium	124 (L)	136 - 145 mmol/L
Potassium	3.9	3.5 - 5.1 mmol/L
Chloride	75 (L)	96 - 111 mmol/L
CO2 total	36 (H)	22 - 30 mmol/L
Anion gap	13	4 - 13 mmol/L
Calcium	10.8 (H)	8.5 - 10.0 mg/dl
BUN	23	8 - 25 mg/dl
Creatinine	0.86	0.75 - 1.35 mg/dl
BUN/creatinine ratio	27 (H)	6 - 22
Albumin	3.6	3.5 - 5.0 g/dl
Alkaline phosphatase	707 (H)	45 - 115 U/L
ALT (sgpt)	118 (H)	10 - 55 U/L
AST (sgot)	192 (H)	8 - 45 U/L
Bilirubin total	1.6 (H)	0.3 - 1.3 mg/dl
Bilirubin direct	0.3	0.1 - 0.4 mg/dl
Protein total	8.2	6.4 - 8.3 g/dl
Ammonia	24	15 - 50 umol/L
Prothrombin time	18.7 (H)	9.1 - 13.9 seconds
INR	1.61 (H)	0.80 - 1.20

Our patient continued to experience symptomatic hypoglycemia over the next several weeks. The cause of the hypoglycemia was likely multifactorial, including an insulin-like growth factor 2 (IGF-2)-related aspect, progression of liver disease, and prolonged corticosteroid exposure (as treatment for transaminitis secondary to his previous chemotherapy regimen). Given his refractory hypoglycemia endocrinology was consulted, and our palliative care team proceeded with coordinating the recommended interventions. He was treated with dexamethasone doses between 6 mg and 8 mg daily, however the addition of 10% dextrose/multiple electrolyte solution IV at rates between 30 to 40 mL/hr was necessary to maintain blood glucose levels above 70 mg/dL. Additional recommendations included the addition of diazoxide, 250 mg twice daily, which treats hypoglycemia through inhibition of insulin release from the pancreas [10]. Throughout, our team kept close communication with the family and hospice agency ensuring this treatment plan would be possible at home. He was ultimately discharged with a succinct bundle of interventions (Table 2) that were able to be administered at home with hospice care to best control his symptoms. The dextrose infusion at a rate of 40 mL/hr worked well logistically for the family and hospice team, as the one-liter bag of fluid could be changed every 24 hours. Due to persistent alerts from the continuous glucose monitor, our team adjusted the monitoring parameters to prevent alarm fatigue, as the lower end of normal cutoff had been set at 70 mg/dL, and his glucose values were persistently between 50 to 60 mg/dl. Despite all these interventions, our patient’s symptoms continued to progress while at home with new hematemesis, increasing morphine requirements and worsening hypoglycemia.

**Table 2:** Discharge Interventions for Home Hospice Services

Treatment Interventions
Two continuous glucose monitoring devices
Morphine patient-controlled anesthesia pump with a basal rate of 4 mg/hr and 6 mg bolus every 30 minutes as needed to treat pain.
Continuous infusion of 10% dextrose/multiple electrolytes solution at 40 ml/hr to keep blood glucose > 70 mg/dl.
Prednisone 40 mg oral daily to treat hypoglycemia.
Haloperidol 2 mg intravenous every 6 hours as needed to treat agitation and nausea.
Lorazepam 1 mg oral every 6 hours as needed to treat anxiety.
Midazolam 5 mg intranasal as needed to treat seizures.
Ondansetron 4 mg every six 6 hours as needed to treat nausea.

Due to the unique palliative care challenges, the patient’s family requested inpatient symptom management, and our multidisciplinary hospice team agreed that further interventions for palliation in a home setting were not possible. Ultimately, the patient was transitioned to comfort measures only and admitted to general inpatient hospice care about one month after being admitted to home hospice due to increasing pain and the previously noted symptoms. He appeared comfortable per family’s, nurses’ and palliative care providers’ assessments in his final days and hours. He died after 13 days in inpatient hospice care, approximately six weeks after being admitted to hospice, and more than three and a half years after initial cancer diagnosis.

## Discussion

Our patient’s history is unique in several regards, including the patient’s rare hepatic malignancy diagnosis as an adolescent, cancer-directed treatment with a relatively new locoregional technique, and multiple uncommon and challenging disease sequelae (particularly MGOO and recalcitrant hypoglycemia) requiring palliative interventions and multidisciplinary teamwork. The palliative care team was first introduced to the patient three years after his diagnosis and about seven months prior to his death. During the initial introduction between the service and patient, his symptoms were well controlled and exclusively due to side effects from his chemotherapy. This early involvement of the palliative care team during a time when the patient was not critically ill was crucial for establishing rapport with the patient and his family and allowed for a smooth transition when the palliative interventions were urgently needed. The palliative medicine provider was a familiar physician to the family, and she was able to integrate with the pediatric oncology team, with the important role of delivering the difficult news regarding lack of further treatment options. 

Beyond the pathology and interventions presented in this case, the patient's age of 17 to 20 years old is unique in the field of hospice and palliative medicine. The challenge with this population is that both pediatric and adult palliative care physicians have less experience with this age group - the pediatric group typically sees much younger, while the adult-focused providers tend to see much older patients. About 15% of all consults for palliative care at pediatric-focused hospitals found were for young adults older than 18 years old, while adult-focused palliative care found most patients to be between 50 and 75 years old [11,12]. Addressing this gap in the training of palliative care physicians must be a focus for programs moving forward. 

Malignant gastric outlet obstruction is a serious complication in advanced intra-abdominal malignancies, and most commonly reported in pancreatic, gastric, colorectal and duodenal malignancies [13]. The most recent guidelines from the American Society for Gastrointestinal Endoscopy recommend palliative intervention with endoscopic ultrasound guided gastroenterostomy (EUS-GE, of which EUS-GJ is one form) rather than surgical gastroenterostomy for incurable MGOO, particularly in patients who value early discharge and resumption of oral diet [13]. For our patient, prior to this intervention he was unable to tolerate any oral intake. This procedure allowed him to tolerate a regular diet, be at home and we suspect extended his life by several weeks.

Initial symptoms of hypoglycemia include shakiness, tachycardia, anxiety, and diaphoresis, and neurologic symptoms of confusion, amnesia, psychosis, seizures, and coma, which can result in death [14,15]. Beyond the direct effect of the tumor consuming glucose, the destruction of healthy hepatic cells limits the physiologic functions of gluconeogenesis and glycogen breakdown. The pathophysiology of hypoglycemia is likely multifactorial and theorized to be due to NICH, which is characterized by secretion of IGF-2 by the tumor [14]. IGF-2 mimics insulin, causing increased glucose uptake by skeletal muscle tissue, decreased hepatic glucose production and suppression of the counter-regulatory hormones of growth hormone, cortisol and insulin-like growth factor 1 [14,16,17]. Diagnosis can be challenging typically after ruling out other causes such as endocrinopathies, sepsis, organ failure, or starvation. Evaluation should focus on diagnosing insulin-related hypoglycemia, and if low endogenous insulin is present, then pro-IGF-2 should be measured [14]. Given the pathophysiology described above, the most critical treatment is surgical resection of the tumor [18]. The first line pharmacologic treatment is glucocorticoids which increase gluconeogenesis and reduce peripheral utilization of glucose [18]. Prednisolone may be preferred to prednisone as it is the active metabolite and does not require hepatic conversion. Diazoxide is prescribed to treat hyperinsulinism as it inhibits insulin release and can be utilized in NICH to counteract the effects of IGF-2. Other pharmacologic options include glucagon, growth hormone and somatostatin analogues. Our case is unique due to the component of adrenal insufficiency and steroid withdrawal after treating a side effect of chemotherapy, as well as utilizing home hospice services for home infusion of dextrose replacement daily.

## Conclusions

In conclusion, our case demonstrates the importance of establishing palliative care early in a patient's disease. Our relationship enabled value-based interventions to be provided for challenging complications in advanced cancer. The palliative treatment of MGOO with EUS-GJ allowed for a minimally invasive procedure to be performed at a time of crisis to quickly address symptoms, and allow the patient to return to eating by mouth and get back to his home. Utilizing home hospice services for continuous dextrose infusions allowed our patient symptomatic control of his severe and persistent hypoglycemia and kept him in the setting he wished for at the end of his life. 
